# Obesogenic Environment in a Minas Gerais State Metropolis, Brazil: Analysis of Crime Rates, Food Shops and Physical Activity Venues

**DOI:** 10.3390/ijerph21121700

**Published:** 2024-12-20

**Authors:** Monique Louise Cassimiro Inácio, Luana Caroline dos Santos, Olívia Souza Honório, Rafaela Cristina Vieira e Souza, Thales Philipe Rodrigues da Silva, Milene Cristine Pessoa

**Affiliations:** 1 School of Nutrition, Federal University of Ouro Preto, Ouro Preto 35400000, Brazil; monique.louise0607@gmail.com (M.L.C.I.); olivia.honorio@aluno.ufop.edu.br (O.S.H.); 2 Nursing School, Federal University of Minas Gerais, Belo Horizonte 30130100, Brazil; luanacstos@gmail.com (L.C.d.S.); rafasouzacec@gmail.com (R.C.V.e.S.); 3 Paulista School of Nursing, Federal University of São Paulo, São Paulo 04021001, Brazil; thalesphilipe27@hotmail.com

**Keywords:** built environment, obesogenic pattern, environmental analysis

## Abstract

The aim of the present study is to identify obesogenic environment profiles to find the obesogenic environment pattern for Belo Horizonte City. The current research followed the ecological approach and was substantiated by data from food shops, public sports venues, crime rates (homicides and robberies) and the rate of accidents with pedestrians. Descriptive analyses and principal component analysis (PCA) were conducted in Stata software, version 14.0. Georeferencing and map plotting were carried out in Qgis software, version 2.10. All neighborhoods in Belo Horizonte City (n = 486) were included in the study. The obesogenic pattern comprised the highest mean number of shops selling ultra-processed food, crime rates, and accidents with pedestrians. The generated latent variable was divided into tertiles, and the second and third tertiles represented the most obesogenic environments. Neighborhoods accounting for the highest obesogenic profile also recorded the largest number of shops selling all food types. Furthermore, neighborhoods in the third tertile recorded the highest mean income (BRL 2352.00) (*p* = 0.001) and the lowest Health Vulnerability Index (HVI = 54.2; *p* = 0.001). These findings point towards the need for developing actions, policies and programs to improve these environments, such as tax incentives to open healthy food retailers and public sports venues to promote healthier lifestyles and to prevent diseases in the middle and long term.

## 1. Introduction

Urban determinants, such as access to healthy food, infrastructure for physical activities, public security and quality of public transportation are factors influencing the population’s lifestyle and well-being [1]. Environmental aspects have been having stronger impact on the health–disease binomial over time. Neighborhoods accounting for the largest number of shops selling ultra-processed food, for the lowest degree of walkability and for the lowest public security rates are associated with obesity development [2,3,4]. According to predictions by the World Obesity Federation, 51% of the world population—i.e., more than 4 billion people—will be overweight or obese within the next 12 years. Currently, approximately 63% of the Brazilian population is overweight. Moreover, 57.9% of Belo Horizonte’s population is overweight [5].

From this perspective, SDG 11 is a UN goal aimed at making cities and human settlements inclusive, secure, resilient and sustainable. This goal is one of the 17 Sustainable Development Goals (SDGs) created by the UN in 2015. SDGs are part of a global agenda focused on elaborating and implementing public policies to eradicate poverty, preserve the environment, avoid climate change and make sure that the global population can have good quality of life [6]. Accordingly, acknowledging the influence of urban environments on the physical, mental and social health of city residents is essential to build healthy and sustainable cities [7,8]. The association between this goal and the obesogenic environment is clear because urban environments and planning policies can have a direct influence on eating behaviors and physical activities, which are crucial factors to prevent obesity [5,9].

In addition to the aforementioned requirements, healthy and sustainable cities must ensure the availability of, and access to, healthy food [10]. Houses close to shops that sell healthy food are the first step to understand the influence of the environment on eating practices. In 1997, New Zealand researchers introduced the term “obesogenic environment”, which regards a set of environmental aspects leading to unhealthy eating practices and to sedentary lifestyles. The obesogenic environment is one of the determining factors for overweight and obesity development and, consequently, to the development of other morbidities, such as diabetes, hypertension and hypertriglyceridemia [11].

Thus, acknowledging the territory from the perspective of individuals living in them is essential to identify potentially obesogenic environments as part of their diagnosis and to develop actions to help build assertive care practices linked to health and nutrition [12].

The environmental approach linked to healthcare and nutrition practices is linked to the relevance of having spaces capable of allowing physical activity and of providing access to healthy food. These factors are determining for both good health conditions and food consumption since they have a direct impact on the population’s nutritional status [13].

Accordingly, it is important to develop in-depth studies on environmental featuring the support of actions aimed at forming neighborhoods that encourage healthier lifestyles and, consequently, that prevent adverse health outcomes. Based on jointly assessing these factors, given the limited evidence and interventions focused on environments’ obesogenic potential, the aim of the present study was to describe the obesogenic environment of neighborhoods in Belo Horizonte City in order to develop an obesogenic environment pattern.

## 2. Materials and Methods

### 2.1. Study Design

An ecological study was carried out with data for the food environment, an environment good for physical activities, and the social environment. Belo Horizonte is the capital of Minas Gerais State. It is the sixth most populous city in the country, with 2,530,701 inhabitants. The city, which is located in Southeastern Brazil, recorded a Human Development Index (HDI) of 0.810 and demographic density of 7167 inhabitants/km^2^ [14].

### 2.2. Environmental Data

Environmental variables were chosen to be included in the study because it is well known that obesogenic environments encompass numerous social and built environment aspects, such as the availability of food shops, green areas, equipment for physical activity and public security [1]. From this perspective, studies have suggested that socioeconomically vulnerable neighborhoods tend to have lower availability of healthy food than those accounting for higher purchasing power [2,15,16]. Thus, there may be lower fruit, vegetable and legume consumption in these neighborhoods, as identified in a study conducted by Menezes et al. [17], according to whom individuals living in places with low availability of shops that sell fresh food tend to consume lower amounts of this food type. In addition, social environment features, such as crime rates in the neighborhood, can have impact on weight gain due to lack of public security for going out to exercise, a fact that can contribute to overweight and obesity development [18]. Some research has shown [4,19] that individuals who perceive their neighborhoods as unsafe are more likely to present excess weight. Pedestrians are seen in public spaces in safer places and it can encourage personal and social interactions and favor the practice of physical activities, such as walking, jogging, and outdoor sports [4].

#### 2.2.1. Retail Food Environment Data

Data related to the food retail environment from 2019 were made available by the State Secretariat of Finance of Minas Gerais. They listed formal food sales shops, according to the National Classification of Economic Activities (NCEA). This instrument is applied to codify the national standardization of economic activities and to frame criteria used by several tax administration bodies in Brazil [20].

Neighborhoods in Belo Horizonte City were herein adopted as analysis units. Food sales shops were gathered to feature the food retail environment based on the classification by the Technical Study of Mapping Food Deserts in Brazil [21]. This classification combines three aspects: (1) core activity of a given shop based on the NCEA, (2) food types most often sold in shops based on data in the 2008/2009 Family Budget Survey (POF), and (3) categorizing the most often sold food types, according to NOVA classification [22]. Food-selling shops were classified into three categories: “natural food purchasing shops”, “mixed shops” and “ultra-processed-food-selling shops”. Fresh-food-selling shops, where fresh food represents more than 50% of the total amount of sold food, include fruit and vegetable shops, butchers and fishmongers. Mixed-type shops are those where there is no prevalence of a specific food type, such as restaurants, bakeries, dairies, grocery stores, supermarkets and hypermarkets. Ultra-processed-food-selling shops are those where these food types account for more than 50% of all sold food, such as cafeterias, candy stores and bars [21].

It is essential to notice that ultra-processed food is based on industrial formulations prepared with few whole foods or with no whole food at all. It is based on ingredients such as additives, dyes and preservatives, which are used to change flavor, color and texture. Ultra-processed food has high levels of calories and is poor in nutrients. Snacks, soft drinks, cookies and fast food are examples of it. On the other hand, fresh food (in natura) comes right from plants or animals, and it is not subjected to any processing after its collection. Fruits, vegetables, fresh meat and eggs are good examples of fresh food. These food types are consumed as harvested or bred and present preserved nutritional features. Minimally processed food, in its turn, undergoes processes free from the addition of any substance, only regular preparations for its consumption, such as washing, freezing or grinding. These food types keep most of their original nutritional qualities. Dried grains, pasteurized milk and frozen vegetables are good examples of these food types. Finally, processed foods undergo preservation processes based on salt, sugar and oil addition, among other substances. These processes extend food shelf life but change its flavor and texture; however, the food keeps part of its original ingredients. Bread and cheese are good examples of these food types [22].

The ratio of shops selling fresh and minimally processed food was calculated based on the ratio of these shops and the sum of mixed shops and of shops selling ultra-processed food and shops selling fresh and minimally processed food, multiplied by 100. The ratio of mixed shops was calculated based on the ratio of these shops and the sum of shops selling fresh and minimally processed food and of shops selling ultra-processed and mixed food, multiplied by 100. Density was calculated based on the ratio between the number of shops and the total population, multiplied by 5000, as proposed by Justiniano et al. [23].

#### 2.2.2. Social Environmental Data

The social environment was analyzed based on the number of crimes (homicides and robberies) and accidents with pedestrians. These data were provided by Minas Gerais State Secretariat of Public Security and they also regard the total number of police reports in a given neighborhood in 2019.

#### 2.2.3. Environmental Data on Physical Activity

Data related to environments good for exercising were provided by Belo Horizonte City Hall. The following public sports venues were included: cycling routes, walking trails, health gyms, outdoor gyms, parks and squares. The addresses of these locations were collected and georeferenced.

#### 2.2.4. Obesogenic Environment Pattern

The following environmental variables were taken into consideration to identify the “obesogenic environment pattern”: number of shops selling ultra-processed food, rate of crimes (homicides and robberies) and of accidents with pedestrians, and public sports venues (cycling routes, walking trails, health gyms, outdoor gyms, open spaces and squares) recorded for all 486 neighborhoods in Belo Horizonte City. The score generated from statistical analyses was divided into tertiles; the second and third tertiles represented the most obesogenic patterns.

#### 2.2.5. Georeferencing

Georeferencing was carried out by electronically capturing the addresses of food-selling shops, public sports venues and neighborhoods where the largest number of crimes and accidents with pedestrians was recorded. This list was elaborated in a Geographic Information System (GIS). Data were treated in the WGS 84 Geographic Coordinate System (latitude and longitude) and turned into a Projected Coordinate System, Universal Transverse Mercator System (UTM), 23S time zone and SIRGAS 2000 datum in QGIS software, version 2.10.4.

#### 2.2.6. Data Analysis

The data analysis was conducted in 2024. The obesogenic environment pattern was identified through principal component analysis (PCA). This is an exploratory analytical method used to gather information about the observed variables turned into a smaller number of variables while causing minimal information loss [24].

The following variables were adopted to conduct the PCA: rate of crimes (homicides and robberies) and accidents with pedestrians, public sports venues (cycling routes, walking trails, health gyms, outdoor gyms and squares) and the mean number of shops selling ultra-processed food in all Belo Horizonte neighborhoods.

The Kaiser–Mayer–Olkin (KMO) value was estimated as a PCA adequacy measurement. Values ranging from 0.5 to 1.0 were considered acceptable for this index [25]. Subsequently, components recording values > 1.0, based on the scree plot, were excluded from the PCA. Factor ‘loading’ higher than 0.4 and *p* < 0.05 in the analysis of standardized estimates applied to build latent variables highlighted that the magnitude of the correlation between the observed variable and the constructor was moderately high [25].

The categorized maps were plotted to identify the obesogenic profile of the assessed neighborhoods in QGIS software, version 2.10. Darker colors correspond to neighborhoods with a stronger obesogenic profile. Neighborhoods presenting lighter colors are featured as less obesogenic.

Descriptive analysis was carried out based on absolute and relative frequency calculations, central tendency measurements and dispersion. These procedures allowed featuring the neighborhoods based on their obesogenic profile. The Chi-square test was adopted to compare the ratios and the means and medians. The Kruskal–Wallis test was applied and it was followed by Tukey’s post hoc test to identify the significant pair. All tests were run at 5% significance level (*p* < 0.05). All statistical analyses were conducted in Stata software, version 14.0.

#### 2.2.7. Ethical Aspects

The present study was approved by the Ethics and Research Committee of Federal University of Minas Gerais (UFMG), under Opinion n. ETIC 0079.0.203.000-10 and 52537215.5.0000.5149.

## 3. Results

**Figure 1:**
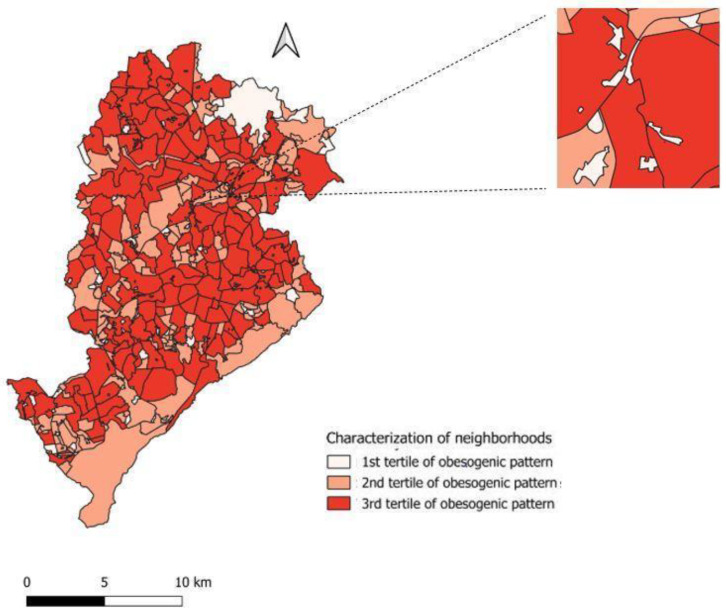
Map of Belo Horizonte neighborhoods’ features based on the obesogenic pattern found through PCA analysis.

The main component formation was identified and it contributed to 69.4% of the variance in total information (Table 1). The KMO index was 0.624 and the factor loading of all variables was satisfactory (>0.40), except for variable “public sports venues”, which reached 0.218. The explained variance recorded for the formed principal component increased to 89.6% after this variable was excluded, and the KMO index reached 0.705. All variables (shops selling ultra-processed food, accidents with pedestrians and rate of crimes) forming the latent variable “obesogenic environment” presented satisfactory factor loading and positive values. Finally, the latent variable generating the “obesogenic environment” was categorized into tertiles; the second and third tertiles were the most obesogenic ones (Figure 1).

Neighborhoods in the third tertile recorded the highest density and ratio of shops selling all food types in the analysis, unlike neighborhoods presenting the lowest obesogenic profile, which accounted for the lowest density and ratio of shops selling all food types, as shown in Table 2.

Table 3 presents the rate of neighborhoods in each administrative region classified based on the first, second and third obesogenic profile tertile. No significant difference was found between regions, and this finding has evidenced that Belo Horizonte City presents a homogeneous obesogenic pattern. Approximately 13% of neighborhoods in the first tertile are located in Western Belo Horizonte City, 13.58% of neighborhoods in the third tertile are located in its northeastern region and 13.58% of neighborhoods in the Barreiro region are also in the third tertile.

With respect to mean income in the neighborhoods and to HVI, neighborhoods with stronger obesogenic profiles also account for the highest incomes and for the lowest HVI, as shown in Table 4.

## 4. Discussion

The study findings point towards an obesogenic environment pattern comprising a larger number of shops selling ultra-processed food, as well as crimes and accidents with pedestrians. Neighborhoods accounting for the strongest obesogenic profile recorded the highest density and ratio of shops selling all food types, the highest mean incomes and the lowest HVI.

Casey et al. [26] emphasized that environmental factors are complex and interact in different ways; therefore, it is essential to take into consideration the impact of the combined presence or absence of the assessed variables, rather than assessing the impact of each one of these variables separately. However, this is not what was observed in the present research, since there was little evidence featuring the environments based on multifaceted perspectives. Most analyses have taken into account different obesogenic environment aspects separately in order to assess the food retail environment, good environments for physical activities or the social environment [4,27,28]. This scenario made it difficult to compare results from different studies [29].

When it comes to assessing obesogenic environment components separately, a study conducted in Ouro Preto City, Minas Gerais State, showed a larger number of shops selling all food types in central census tracts located in tertiles accounting for the highest income per capita and for the lowest social vulnerability. The same scenario was not observed in peripheral areas, where there was a smaller number of shops, mainly of those selling healthy food—the same profile is observed in other Brazilian cities [28,30,31]. With respect to physical activities and the social environment, studies have suggested that the presence of pedestrians in public spaces, in safer places, can encourage personal and social interactions, as well as favor the practice of physical activities, such as walking, jogging and outdoor sports [4,18,19].

From this perspective, promoting healthy and sustainable cities is a crucial issue to guarantee social well-being. The term ‘sustainable city’ can be understood as a city seeking to balance urban growth and economic development in order to achieve environmental preservation, social justice and residents’ quality of life. Some fundamental principles linked to these cities encompass quality of air and water, social inclusion, access to health services, public security and sustainable urban planning, in addition to access to both healthy food and public transportation, among others [8,10]. Sustainable urban planning can reduce obesogenic factors by increasing access to fresh food, encouraging local markets and urban agriculture. In addition, creating parks, bike paths and safe recreational spaces encourages physical activity practice, whereas well-lit streets and accessible sidewalks increase safety for walking and cycling. Policies limiting the presence of fast food outlets near schools also promote healthier food choices [32].

It is necessary to understand that an individual’s lifestyle is not random. It results from everyday life in a given environment. This process also results from supply chains. This perspective is linked to several life practices observed across places and territories. These practices influence individuals’ health conditions, as well as that of their family members and community [12].

The aim of healthy and sustainable cities is to ensure easy access to adequate food, such as fruits, vegetables and fresh products. In addition, they encourage exercising in public spaces, such as parks, cycling routes and squares. Efficient public transportation systems and secure environments for pedestrians are also factors deserving attention when it comes to exercising. It is so because they encourage people to walk and to use public transportation, which promotes a more active lifestyle. Cities where people rely on private transportation means face challenges related to lack of exercising. It is also important to highlight that security to residents in public spaces is a relevant component to fight sedentary lifestyles, since it allows people to feel safe to exercise and to socialize in public spaces in their communities [7,8,10].

The current results showed that neighborhoods featured in the third obesogenicity tertile accounted for higher income and lower HVI. However, the literature shows that lower-income locations face major obstacles when it comes to promoting healthy environments [33]. In many cases, low-income communities face barriers, such as limited access to healthy food, lack of safe spaces for physical activities and higher concentration of shops like small markets and bars, with little fresh food supply [34]. These aspects reflect not only a public health issue but also a matter of social and environmental justice. Everyone should have equitable access to environments that promote health and well-being, regardless of their location or economic status [35]. However, reality often shows the opposite. These inequalities are reinforced by urban policies, market structures and, most of the time, by lack of these communities’ representation in decisions that have impact on the environment they live in [33].

Lack of access to information about informal shops available in governmental databases is a limitation of the current study. This scenario made it impossible to assess the informal food retail environment. This situation could be neutralized through on-site observation, which proved unfeasible due to logistical and financial issues if one bears in mind the large territorial dimension of the assessed municipality. Findings in the present research helped to acknowledge the territories by featuring their obesogenic environments and their food shops—although they were not taken into account in the present study. Therefore, the study can help contextualizing food and nutrition care practices in order to build healthier and more sustainable cities, in addition to supporting future studies on this topic.

## 5. Conclusions

The observed obesogenic pattern included a higher concentration of shops selling ultra-processed food, a larger number of accidents with pedestrians and a higher rate of crimes. Neighborhoods with stronger obesogenic profiles accounted for the highest income rates and for the lowest HVI values in comparison to those recording the lowest obesogenic profile.

These findings highlight the need for urgent actions, policies and programs to improve these environments, which would include zoning regulations to limit ultra-processed food outlet concentrations, mainly in areas close to schools, hospitals and homes, and promoting spaces for local fresh food markets. This target can be reached through urban zoning policies and commercial establishment licensing. This action favors access to healthy and affordable food, in compliance with SDG 11, which seeks to promote healthier and more sustainable urban environments. In addition, it is necessary to invest in urban infrastructure to ensure accessible sidewalks, well-lit parks and bike paths, as well as to increase public security in high-risk areas. These actions encourage regular physical activity and create a safer environment for citizens, which are also features in compliance with SDG 11 because they promote safe and inclusive urban spaces.

## Figures and Tables

**Table 1:** Factor loadings of variables “Unhealthy establishments”, “Hit-and-runs”, “Criminality”, “Public location for sports practicing”, which form the latent variable in the principal component analysis applied to pattern formation.

Indicators	Analysis 1	Analysis 2
Unhealthy establishments *	0.565	0.563
Hit-and-runs **	0.553	0.574
Criminality ***	0.571	0.593
Public location for sports practice ^&^	0.218	-
Eigenvalue	2.776	2.688
Explained variance (%)	69.4	89.6
Kaiser–Meier–Olkin (KMO)	0.624	0.705

* Number of shops selling unhealthy food (sum of bars, snack bars and candy stores) per neighborhood in 2019. ** Number of hit-and-runs per neighborhood in 2019. *** Total number of homicides and robberies per neighborhood in 2019. ^&^ Number of public locations for sports practicing (sum of walking trails, bike paths, open-air gyms, health gyms and squares) in the neighborhood in 2019.

**Table 2:** Density ratio per 5000 inhabitants, density ratio of shops selling ultra-processed foods/shops selling fresh (in natura) and minimally processed food in Belo Horizonte City, based on obesogenic pattern.

Categories	Obesogenic Pattern			*p* Value
	1st Tertile	2nd Tertile	3rd Tertile	
Index of shops mainly selling fresh and minimally processed foods	8.75 ^a^	9.87	10.33 ^a^	0.001
Index of mixed shops	41.09 ^a^	44.72 ^a^	43.89	0.001
Density of shops mainly selling fresh and minimally processed food per 5 thousand inhabitants	3.00 ^a^	3.93 ^b^	6.39 ^a,b^	0.015
Density of mixed shops per 5 thousand inhabitants	14.42 ^a^	18.85 ^b^	27 ^a,b^	0.001
Ratio of density of shops selling ultra-processed foods/shops selling fresh and minimally processed foods	0.19 ^a^	0.24	0.25 ^a^	0.001

Lowercase letters show statistical differences. a or b only in lowercase means significant statistics. a and b together in lowercase means that values were statistically different from more than one category. Significance value (*p* < 0.05).

**Table 3:** Ratio of neighborhoods with different obesogenic patterns in Belo Horizonte City (MG), per administrative region.

	Administrative Region									
Obesogenic Pattern	Northern	Southern-Central	Northeastern	Barreiro	Western	Northwestern	Pampulha	Venda Nova	Eastern	*p* Value
1st tertile	5.56 (9)	9.88 (16)	12.96 (21)	17.90 (29)	13.58 (22)	10.49 (17)	12.96 (21)	9.26 (15)	7.41 (12)	0.66
2nd tertile	11.73 (19)	11.11 (18)	13.58 (22)	17.90 (29)	11.73 (19)	8.02 (13)	16.67 (27)	6.17 (10)	3.09 (5)	
3rd tertile	11.73 (19)	9.26 (15)	13.58 (22)	13.58 (22)	11.73 (19)	9.88 (16)	12.35 (20)	10.49 (17)	7.41 (12)	

**Table 4:** Health Vulnerability Index (HVI) and mean income based on the obesogenic profile of neighborhoods in Belo Horizonte City (MG).

Category	Obesogenic Pattern			
	1st Tertile	2nd Tertile	3rd Tertile	*p* Value
Mean income	847.38 (0–5915) ^a^	1544.59 (0–11,901)	2352 (0–15,812) ^a^	0.001
HVI				
Low	18.3 (20) ^a^	27.5 (30)	54.2 (59) ^a^	0.001
Moderate	33.0 (76)	33.5 (77)	33.5 (77)	
High	37.8 (56)	33.8 (50)	28.4 (42)	
Very high	40.0 (18) ^a^	37.8 (17)	22.2 (10) ^a^	

^a^ differtatistically significant.

## Data Availability

No new data were created or analyzed in this study.
